# Phylogenomics recovers multiple origins of portable case making in caddisflies (Insecta: Trichoptera), nature’s underwater architects

**DOI:** 10.1098/rspb.2024.0514

**Published:** 2024-07-03

**Authors:** Paul B. Frandsen, Ralph W. Holzenthal, Marianne Espeland, Jesse Breinholt, Jessica A. Thomas Thorpe, Sabrina Simon, Akito Y. Kawahara, David Plotkin, Scott Hotaling, Yiyuan Li, C. Riley Nelson, Oliver Niehuis, Christoph Mayer, Lars Podsiadlowski, Alexander Donath, Bernhard Misof, Emily Moriarty Lemmon, Alan Lemmon, John C. Morse, Shanlin Liu, Steffen U. Pauls, Xin Zhou

**Affiliations:** ^1^ Department of Plant and Wildlife Sciences, Brigham Young University, Provo, UT, USA; ^2^ Department of Entomology, University of Minnesota, St Paul, MN, USA; ^3^ Museum Koenig Bonn, Leibniz Institute for the Analysis of Biodiversity Change (LIB), Bonn, Germany; ^4^ Rapid Genomics, Gainesville, FL, USA; ^5^ Wellcome Sanger Genome Institute, Hinxton, Cambridge, UK; ^6^ Rosenheim University of Applied Sciences, Rosenheim, Germany; ^7^ McGuire Center for Lepidoptera and Biodiversity, Florida Museum of Natural History, University of Florida, Gainesville, FL, USA; ^8^ Entomology and Nematology Department, University of Florida, Gainesville, FL, USA; ^9^ Department of Biology, University of Florida, Gainesville, FL, USA; ^10^ Department of Watershed Sciences, Utah State University, Logan, UT, USA; ^11^ Institute of Plant Virology, Ningbo University, Ningbo, Zhejiang Province, People’s Republic of China; ^12^ Department of Biology, Brigham Young University, Provo, UT, USA; ^13^ Department of Evolutionary Biology and Ecology, Institute of Biology I (Zoology), University of Freiburg, Freiburg, Germany; ^14^ Rheinische Friedrich-Wilhelms-Universität Bonn, Bonn, Germany; ^15^ Department of Biological Science, Florida State University, Tallahassee, FL, USA; ^16^ Department of Scientific Computing, Florida State University, Dirac Science Library, Tallahassee, FL, USA; ^17^ Department of Plant & Environmental Sciences, Clemson University, Clemson, SC, USA; ^18^ Department of Entomology, College of Plant Protection, China Agricultural University, Beijing, People’s Republic of China; ^19^ LOEWE Center for Translational Biodiversity Genomics (LOEWE-TBG), Frankfurt, Germany; ^20^ Senckenberg Research Institute and Natural History Museum Frankfurt, Frankfurt, Germany; ^21^ Department of Insect Biotechnology, Justus-Liebig-University Gießen, Gießen, Germany

**Keywords:** phylogenomics, caddisfly, silk, aquatic insects, Trichoptera

## Abstract

Caddisflies (Trichoptera) are among the most diverse groups of freshwater animals with more than 16 000 described species. They play a fundamental role in freshwater ecology and environmental engineering in streams, rivers and lakes. Because of this, they are frequently used as indicator organisms in biomonitoring programmes. Despite their importance, key questions concerning the evolutionary history of caddisflies, such as the timing and origin of larval case making, remain unanswered owing to the lack of a well-resolved phylogeny. Here, we estimated a phylogenetic tree using a combination of transcriptomes and targeted enrichment data for 207 species, representing 48 of 52 extant families and 174 genera. We calibrated and dated the tree with 33 carefully selected fossils. The first caddisflies originated approximately 295 million years ago in the Permian, and major suborders began to diversify in the Triassic. Furthermore, we show that portable case making evolved in three separate lineages, and shifts in diversification occurred in concert with key evolutionary innovations beyond case making.

## 1. Background

Freshwater is one of the world’s most valuable and limited resources. It is inextricably linked to the survival and diversity of a multitude of terrestrial and freshwater species. Roughly 10% of approximately 1 million described insects spend at least one life stage in water [1], and insects have transitioned from terrestrial to freshwater habitats on nearly 50 occasions [2]. Inhabiting rivers and lakes on six continents, aquatic insects play critical roles in community ecology and ecosystem services [3]. In addition, many species are differentially sensitive to pollution and environmental perturbation and are used to measure the health of aquatic ecosystems [4]. Five insect orders are almost exclusively aquatic (i.e. they require water for their entire larval development): Ephemeroptera (mayflies), Megaloptera (alderflies, dobsonflies and fishflies), Odonata (dragonflies), Plecoptera (stoneflies) and Trichoptera (caddisflies). Of these, caddisflies are the most species-rich, comprising more than 16 000 species [5,6], making them the second most species-diverse radiation among freshwater animals, only behind aquatic Diptera [2,7].

In addition to being diverse, caddisflies are perhaps the most common underwater architects. Most caddisfly larvae build composite underwater structures, as either portable cases or fixed retreats, out of local materials, including small fragments of rocks, sticks and leaves [8]. The key evolutionary adaptation underlying case and retreat building in caddisflies is their ability to produce silk from larval labial glands [9], a character they share with Lepidoptera (butterflies and moths). Trichoptera and Lepidoptera are reciprocally monophyletic [10], and together, they comprise the superorder Amphiesmenoptera. Evidence suggests that the primary molecular components of silk likely evolved in the stem lineage leading to Amphiesmenoptera [11,12]. However, in contrast to the aquatic environments that caddisflies inhabit, almost all Lepidoptera are terrestrial, and both orders use silk that is adapted to their specific environments [13]. For example, retreat makers can dramatically alter the physical environments of aquatic ecosystems, primarily at the interface between flowing water and the streambed. Individuals from the retreat-making family Hydropsychidae frequently occur at densities of thousands per square metre [14], with each larva building its own net and retreat to filter organic particles from flowing water and fill interstitial spaces in the streambed [15]. Additionally, the evolutionary innovation of portable case making has allowed for increased respiratory efficiency and the exploitation of lentic habitats. Cases and retreats also protect larvae from predation both structurally and by camouflage, aid them in maintaining their position in fast-flowing waters and provide a protective pupal structure [3,6,8]. Despite their fundamental importance to the ecology and monitoring of freshwater ecosystems, basic questions about caddisfly evolution, including the origin and diversification of their structures, remain unresolved, limiting our understanding of their unique but highly successful life histories.

Early systematic studies of Trichoptera were primarily based on morphology and were conducted before modern analytical methods became available [16–20]. Recently, studies based on a handful of molecular markers or mitochondrial genomes generated conflicting results [7,21,22]. Despite conflicting results, these studies consistently grouped caddisflies into two major subordinal clades that reflect the extended phenotypes of their larvae: Integripalpia and Annulipalpia. Integripalpia [21] includes portable case makers and free-living caddisflies, while Annulipalpia includes the ‘fixed retreat makers’, whose larvae build fixed structures, usually in flowing waters, on rocks or other submerged substrates. Within Integripalpia, the relationships among five families with varying case-making strategies remain unresolved. These include the predatory, free-living caddisflies in the families Rhyacophilidae and Hydrobiosidae, the tortoise case makers in the family Glossosomatidae and two families of microcaddisflies, Hydroptilidae and Ptilocolepidae. These families have previously been included in a group called ‘Spicipalpia’ [17,18], ‘closed cocoon-makers’ or simply ‘cocoon-makers’ owing to their construction of semi-permeable closed cocoons [23]. However, most Trichoptera researchers suspect that this group is not monophyletic, as a morphological assessment [24] and all molecular estimates have recovered this grouping as para- or polyphyletic [7,21,22,25,26]. Because these families arose during the early evolution of the order and represent a diversity of silk-use and case-making strategies, understanding their correct placement in the evolutionary history of caddisflies is critical to determining the origin of case making as well as other behaviours of Trichoptera. Additionally, resolving the timing of the origin and diversification of caddisflies is essential for building a better understanding of the innovations that led to the evolutionary success of this critical group of freshwater insects.

To shed light on these questions, we generated a phylogenomic dataset aimed at estimating the evolutionary history of caddisflies. We combined de novo transcriptome sequencing, targeted exon capture sequencing and publicly available genome assemblies to generate a dataset for 207 caddisfly species representing 174 genera and 48 of 52 extant families. We resolve the timing and pattern of the evolutionary history of Trichoptera, including key nodes that have long been a systematic difficulty. In addition, we use this pattern to infer ancestral states of larval retreat and case making, as well as pupal constructions. We then link our phylogenetic results to the evolution of the incredible array of underwater architectures constructed by caddisflies, thereby providing a key link between evolutionary history and phenotypic diversity that impacts the physical structure, hydrology and ecology of freshwaters worldwide.

## 2. Results

### (a) Transcriptome results

Our final alignment in the transcriptome-only dataset consisted of 1 253 378 amino acids from 3206 groups of orthologous genes. The maximum likelihood (ML) tree based on the concatenated and partitioned supermatrix was largely congruent with the species tree generated in ASTRAL, with a few exceptions. Both trees recovered Annulipalpia and Integripalpia as reciprocally monophyletic. As in the study by Thomas *et al*. [21], the five cocoon-making families form a grade leading to the tube case makers, Phryganides (in suborder Integripalpia, *s.l*.). The microcaddisflies (Ptilocolepidae and Hydroptilidae) form a monophyletic group and are sister to the rest of Integripalpia. A clade containing the free-living (Rhyacophilidae and Hydrobiosidae) and tortoise-case-making (Glossosomatidae) caddisflies is then sister to the tube case makers, subterorder Phryganides. While this clade, containing the free-living families and tortoise case makers, has not been recovered in previous phylogenetic analyses, these families have been hypothesized to be closely related based on their pupal behaviour, including the construction of a pupal dome, which protects a silken semi-permeable cocoon [8]. The most notable areas of incongruence between the ASTRAL species tree and the supermatrix-based ML tree were the relationships among these families. In the supermatrix-based tree, the free-living caddisfly family Hydrobiosidae was recovered as sister to a clade containing the free-living family Rhyacophilidae and the tortoise-case-making family Glossosomatidae. However, in the ASTRAL multispecies coalescent (MSC) tree, the two free-living families were recovered as a clade sister to the tortoise case makers. In both cases, the relationships among these families were recovered with lower support than the other relationships in the tree. The ultrafast bootstrap support in the ML tree was 67, while the local posterior probability support [27] in the MSC tree was 0.96 (electronic supplementary material, figure S1). To further investigate this difference, we conducted four-cluster likelihood mapping with permutation tests on the transcriptome-only dataset (electronic supplementary material, figure S2). Four-cluster likelihood mapping recovered a slight preference for (Hydrobiosidae + (Glossosomatidae + Rhyacophilidae)) over the alternative. However, the permutation tests revealed that (Hydrobiosidae + (Glossosomatidae + Rhyacophilidae)) was also the preferred arrangement in permutation tests I and II, revealing that this arrangement may have been driven by bias in the supermatrix-derived tree owing to the non-random distribution of missing data.

### (b) Combined dataset

**Figure 1.**
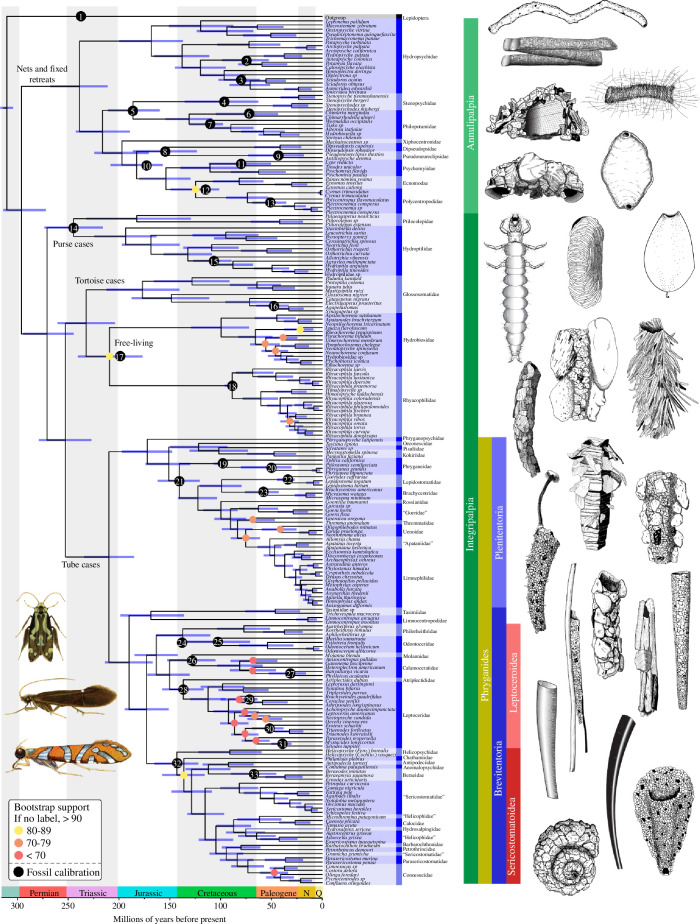
Dated phylogeny of Trichoptera from the combined targeted enrichment and transcriptome dataset. Branches with bootstrap support of less than 90 are labelled with yellow (80–89), orange (70–79) or red (<70) dots. The placement of fossil calibration points is indicated with numbered black dots. Selected larval caddisfly retreats and cases are shown on the right, and selected adult caddisflies are shown on the bottom left. Illustrations by Ralph Holzenthal and Julie Martinez.

To boost the taxon sampling of the transcriptome dataset, we sequenced an additional 158 individuals using targeted DNA enrichment and sequencing [28,29]. After filtering, the final dataset consisted of 339 orthologue groups representing a total of 24 678 aligned amino acids. The deep splits in the ML tree based on the combined partitioned supermatrix were largely congruent with those recovered in the transcriptome-only tree (figure 1). However, contrary to the phylogeny generated from the supermatrix of the transcriptome-only dataset, the tree from the combined dataset recovered the free-living caddisfly families (Rhyacophilidae and Hydrobiosidae) as a monophyletic clade with tortoise case makers (Glossosomatidae) as their sister group. Together they are reciprocally monophyletic with the tube case makers (Phryganides). Four-cluster likelihood mapping on the combined dataset recovered strong support for this arrangement with no evidence of bias in the permutation tests (electronic supplementary material, figure S3).

### (c) Molecular dating of Trichoptera divergence events

We found that the oldest caddisflies in crown Trichoptera were present approximately 295 million years ago in the early Permian (figure 1). All extant higher groups within the order, however, began to diversify in the Triassic, including Annulipalpia (fixed retreat makers), Hydroptiloidea (purse case makers), Rhyacophiloidea (free-living and tortoise case makers) and Phryganides (tube case makers) (electronic supplementary material, table S7). Notably, tube-case-making caddisflies within the infraorder Plenitentoria, the group that includes multiple lineages that construct their cases from flowering plant matter, originated in the mid-Jurassic approximately 170 million years ago [30].

### (d) Ancestral state reconstruction and species diversification analysis

**Figure 2.**
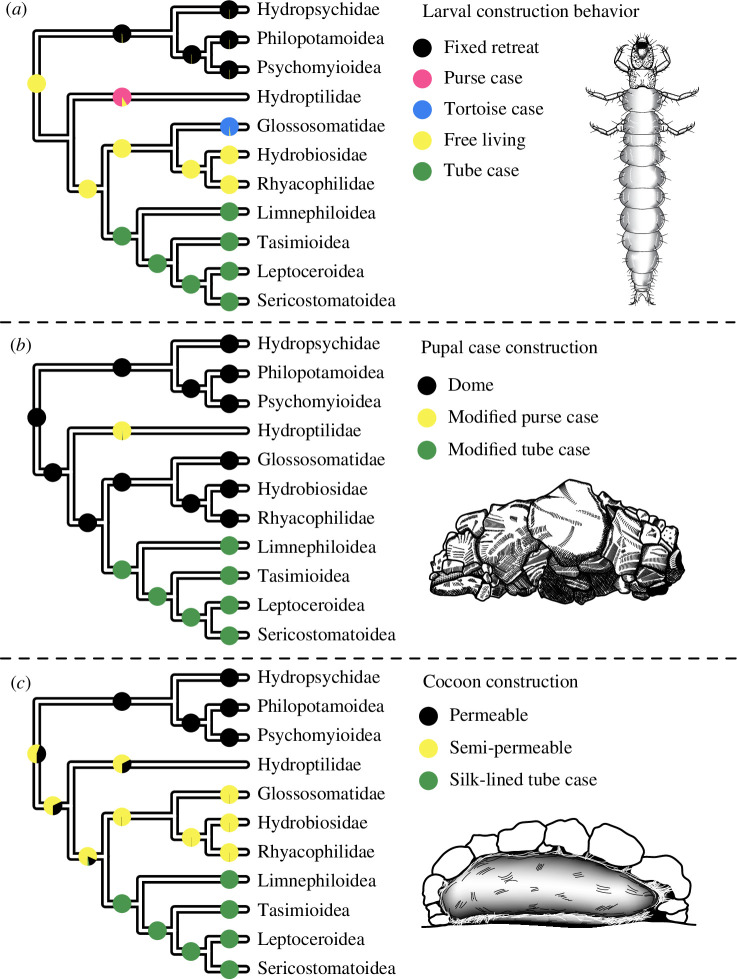
Results of ancestral state reconstruction (ASR) analyses, showing the ancestral states for (*a*) larval construction behaviour, (*b*) pupal case construction and (*c*) cocoon construction. Trees are simplified to major splits for visualization. Only three of the five character states in the pupal case construction ASR are present (with probability > 0.001) in the nodes visible in the simplified tree presented here. Illustrations by Ralph Holzenthal.

We conducted ancestral state reconstructions on both the combined and transcriptome-only datasets to estimate the ancestral states for case-making and cocoon-making behaviour in immature caddisflies. We found that the ancestral trichopteran was free-living and constructed a pupal dome (figure 2). We additionally recovered three independent origins of portable case-making behaviour on each of the branches leading to Hydroptiloidea (purse case makers), Glossosomatidae (tortoise case makers) and Phryganides (tube case makers), contrasting with a previous, untested hypothesis that inferred that purse and tortoise cases are homologous [8]. Full lists of proportions are provided in electronic supplementary material, tables S8–S10.

**Figure 3.**
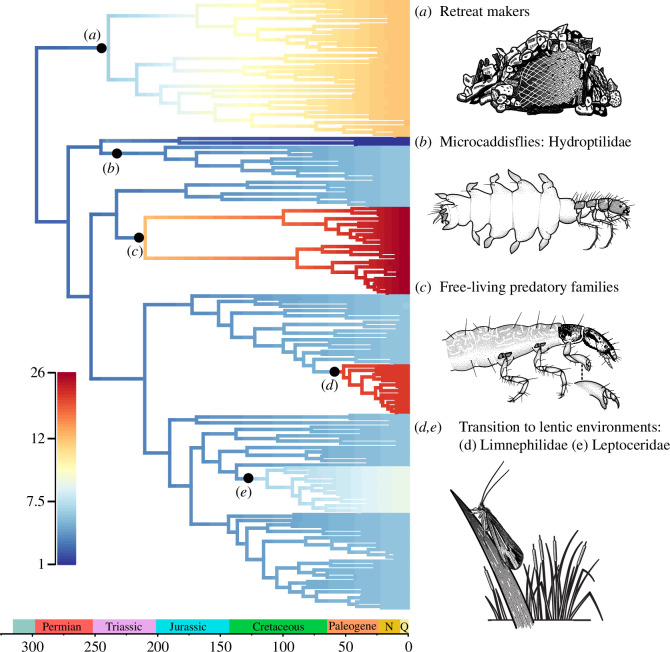
Species diversification rates estimated on the Trichoptera phylogeny, corrected for unequal taxon sampling. Recovered rate shifts are shown in labelled black dots. (*a*) A shift in the lineage leading to the suborder Annulipalpia; (*b*) a shift in the lineage leading to the major family of microcaddisflies, Hydroptilidae; (*c*) a shift in the lineage leading to predatory, free-living caddisfly families Rhyacophilidae and Hydrobiosidae; and (*d*,*e*) two rate shifts that occurred in two tube-case-making lineages that invaded lentic environments. Illustrations by Ralph Holzenthal.

We recovered five shifts in species diversification rate across the caddisfly tree using BAMM [31] on the dated phylogeny from the combined dataset (figure 3). Three occurred during the Triassic and correlated with major groups: the suborder Annulipalpia or fixed-retreat makers; Hydroptilidae, the diverse family of microcaddisflies; and the lineage leading to the predatory free-living caddisfly families Hydrobiosidae and Rhyacophilidae. The other two shifts we recovered are nested within tube case makers on the lineages leading to the diverse families Leptoceridae and Limnephilidae. We also generated a 16 688-taxon tree with stochastic polytomy resolution using Taxonomic Addition for Complete Trees (TACT) [32]. This method allows for the addition of taxa for which there is no molecular information to a phylogeny based on the taxonomic classification of each species alone using birth–death sampling. On this tree, we recovered nearly 30 rate shifts, perhaps owing to the large number of additional taxa (electronic supplementary material, figure S11). While more shifts were recovered on this topology, many shifts coincided with those from the diversification analysis on the phylogeny from the combined dataset (electronic supplementary material, figure S11). The estimated number of transitions from the MiSSE analysis was 7.55. The areas of the tree with elevated diversification rates estimated with MiSSE partially overlapped with those estimated with BAMM (electronic supplementary material, figure S12). In particular, both approaches indicated elevated rates of diversification within the free-living families (Rhyacophilidae and Hydrobiosidae), Limnephilidae and some evidence within Leptoceridae and Hydroptilidae (electronic supplementary material, figure S12). In contrast to the results from the BAMM analysis, MiSSE also recovered elevated rates in a few families within Sericostomatoidea. These discrepancies could be, in part, owing to the different ways that each software accounts for unsampled taxa (clade-specific sampling fractions in BAMM versus a global sampling fraction in MiSSE). The diversification rates estimated across the 25 treePL-dated ML topologies with MiSSE were robust to the variations in tree shape and mirrored those from the diversification rates estimated with the MCMCtree-dated topology.

## 3. Discussion

Trichoptera is the most diverse of the primarily aquatic insect orders and represents the second most diverse freshwater radiation of animals, surpassed only by the aquatic clade of true flies, Culicomorpha/Psychodomorpha [7]. Caddisfly larvae use underwater adapted silk to extend their phenotypes in myriad forms, resulting in underwater architectures that provide camouflage and protection, aid in respiration, facilitate feeding and stabilize the stream beds they inhabit. These extended phenotypes are central to the diversification of the order. The first step towards a better understanding of their diversity is to contextualize the mode of their evolution with a robust phylogeny. Here, we recovered a strongly supported phylogeny for 207 species, representing 48 of the 52 extant families (figure 1). Furthermore, we find that portable case making, responsible for more than half of the diversity of described caddisfly species [5], evolved on three separate occasions from an ancestral free-living form (figure 2). However, despite repeated evolution of portable cases, diversification shifts occurred later, corresponding to other ecological innovations, some made possible only by the earlier use of a portable case (figure 3). However, some of these ecological innovations, such as the invasion of lentic waters, also occurred in other parts of the tree (electronic supplementary material, figure S10) and did not coincide with diversification shifts. These areas of the tree would be excellent candidates for future study into the connections between habitat type and diversification.

The relationships among suborders within Trichoptera have been a point of conflict since the first evolutionary trees were drawn [33–35], pre-dating widespread use of Hennigian systematics. Since then, the primary sources of incongruence among phylogenetic estimates were the relationships among the monophyletic tube case makers (Phryganides), the monophyletic fixed-retreat makers (Annulipalpia) and the five remaining monophyletic families, the free-living families (Rhyacophilidae and Hydrobiosidae), the tortoise case makers (Glossosomatidae) and the two families of purse case-making microcaddisflies (Ptilocolepidae and Hydroptilidae). Multiple arrangements of the relationships among these families were posited, using both morphology and molecular data [7,16–19,21,22,25,26,36]. Thomas *et al*. [21] analysed five nuclear genes and found strong support for the cocoon-making families as a grade leading to the tube case makers, resulting in their reclassification into the suborder Integripalpia. However, the relationships among the families of cocoon makers were not well supported, preventing the interpretation of ancestral states. More recently, an analysis of mitochondrial genomes recovered a similar arrangement with the exception that the microcaddisflies (Hydroptiloidea) were sister to the rest of caddisflies [22]. Here, we estimated a phylogeny with strong support for the grouping of the families of cocoon makers with the tube-case-making families (Phryganides). Furthermore, we found strong support for a clade that includes tortoise case makers (Glossosomatidae) and the two free-living families (Rhyacophilidae and Hydrobiosidae). Although this clade had not previously been recovered with molecular data, morphological experts have long argued that the pupal forms of all three families suggested a common origin [8] and that the tortoise cases of Glossosomatidae likely evolved as ‘precocious pupation behaviour’. Our results are consistent with this interpretation. However, while the monophyly of this clade was strongly supported, the relationships among families within the clade differed among datasets and tree-estimation strategies. Four-cluster likelihood mapping revealed confounding signal in the maximum likelihood analysis of the concatenated supermatrix of transcriptomes, indicating that the relationships recovered in the ASTRAL-III analysis and in the maximum likelihood analysis of the combined dataset, which place Hydrobiosidae as sister to Rhyacophilidae, are more reliable (electronic supplementary material, figure S2). These results are further supported by a recent preprint reporting results from genome-wide sequencing of 29 caddisfly families [37].

Caddisflies began to diversify in the Permian, about 295 million years ago. Our results suggest that the ancestor of all extant caddisflies inhabited the benthos of the oxygen-saturated waters of rivers and streams as a free-living larva, without a portable case or fixed retreat. Like its amphiesmenopteran ancestors, the larva produced silk from labial glands. As it approached pupation, the final instar larva constructed a protective pupal structure of small mineral fragments or other substrates and enclosed itself within the structure in a permeable or semi-permeable silken cocoon. As caddisflies further evolved and diversified, selection favoured the precocious construction of the pupal structure by earlier instars and increasingly complex larval structures. In the suborder Annulipalpia, larvae affixed various materials from the streambed into retreats, offering protection. Eventually, their silk use diversified across the suborder to include capture nets and sophisticated filtration systems associated with their retreats to capture suspended organic particles. In the suborder Integripalpia, owing to the efficacy of the case in providing physical protection, camouflage and more efficient respiration, three different lineages co-opted these precocious pupal structures into portable cases, eventually resulting in purse cases (only in the final instar larvae in Hydroptilidae and Ptilocolepidae), tortoise cases (Glossosomatidae) and tube cases (Phryganides). However, the primary species diversification events in case makers did not coincide with the evolution of the case, but with other ecological innovations (figure 3). For example, bursts in diversification occurred in the lineages leading to the families Leptoceridae and Limnephilidae coinciding with their incorporation of plant matter for case making. Simultaneously, this allowed them to invade slow-moving and standing waters facilitated by the respiratory efficiency of tube cases to enhance the flow dynamics of oxygenated water across the larval abdomen and gills [38]. Since that time, caddisflies have continued to diversify in their respective habitats, eventually becoming one of the dominant animals in freshwater ecosystems.

## 4. Material and methods

### (a) Taxon sampling

As part of the 1K Insect Transcriptome Evolution (1KITE, 1kite.org) project, we generated transcriptome data for 42 new species of caddisflies. We supplemented this dataset with targeted enrichment data for 158 additional museum specimens representing 156 species. We also incorporated a published trichopteran genome [39] and eight previously published transcriptomes (from previous 1KITE papers) into our analyses, resulting in a dataset that comprises 209 in-group taxa, representing 207 trichopteran species, 174 genera and 48 of 52 extant families. We used 18 out-group taxa, including 17 lepidopterans and 1 coleopteran harvested from publicly available transcriptomes and genomes, for a total count of 227 individuals. All details are given in electronic supplementary material, table S1.

### (b) Transcriptome sequencing

We generated 42 new caddisfly transcriptomes and combined them with 8 previously available transcriptomes to create a dataset of 50 species representing 34 families and all of the major clades within the order. Specimens were collected alive and preserved in RNAlater. Specimens were kept cool until RNA extraction. Libraries were prepared and sequenced at BGI-Shenzhen on an Illumina HiSeq 2000. Full details are given in electronic supplementary material, §2.

### (c) Anchored hybrid enrichment probe design and sequencing

Despite extensive efforts to sample and sequence a diverse sample of caddisflies using transcriptomes, our sampling was limited by the species we could collect and identify while alive before destructively preserving them in RNAlater. To expand our taxon sampling to include museum specimens, we used anchored hybrid enrichment (AHE) [28,29]. We used a probe set previously used by Pauls *et al*. [40]. Using this method, we identified approximately 900 exons suitable for phylogenetic inference (electronic supplementary material, §3).

DNA was extracted from museum specimens using a G-biosciences Q-amp extraction kit and sheared to 150–400 bps. Illumina libraries were then prepared following Lemmon *et al*. [29] and combined in pools of approximately 16 samples and enriched. After qPCR-based QC, enriched libraries were sequenced on an Illumina HiSeq 2500 sequencer with a paired-end 150 bp sequencing (electronic supplementary material, §3).

### (d) Transcriptome-only analyses

We followed the 1KITE transcriptome phylogenetic pipeline as described in Kawahara *et al*. [41]. Briefly, transcriptomes were assembled in SOAPdenovo-Trans-63mer v. 1.0.1 (electronic supplementary material, §§4 and 5) [42]. Following transcriptome assembly, we used a set of core orthogroups using the genomes from *Tribolium castaneum*, *Danaus plexippus* and *Bombyx mori* [41] to predict orthologues using Orthograph v. 0.5.4 (electronic supplementary material, §6) [43]. Each resulting orthogroup contained the orthologues of the analysed trichopteran and lepidopteran species. We aligned the amino acids of each orthologue with MAFFT v. 7.475 [44] using the L-INS-i algorithm. We then evaluated the alignments for outliers using the pipeline described in Misof *et al*. [10] (electronic supplementary material, §7). Following alignment, we used Aliscore v. 1.2 (with the option -e enabled) and ALICUT v. 2.3 to identify and remove portions of the alignment that were indistinguishable from random data [45]. Because some gene alignments may not be phylogenetically informative, we used MARE v. 1.2-rc [46] to remove genes with 0 information content. Finally, we generated a concatenated alignment supermatrix using FASconCAT v. 1.0 [47] (electronic supplementary material, §7).

We generated phylogenetic trees using two methods: (i) a ML tree generated from the concatenated supermatrix using partitioned models of molecular evolution using IQ-TREE v. 1.6.7 [48] and (ii) a MSC tree using ASTRAL-III v. 5.7.4 [49]. ASTRAL-III requires individual gene trees as input; thus we generated individual gene trees using ML with IQ-TREE v. 1.6.7 [48]. For each individual gene tree, we selected models of molecular evolution using ModelFinder (-MFP option) as implemented in IQ-TREE v. 1.6.7 and performed 25 separate ML tree searches for each gene. We selected the best tree for each gene and concatenated them for input into ASTRAL-III. We then ran ASTRAL-III using default settings. For the concatenated supermatrix analysis, we first selected an optimal partitioning scheme using the relaxed clustering algorithm, implemented in PartitionFinder 2.0 [50] (electronic supplementary material, §8) [50,51] followed by the selection of protein models for each metapartition using ModelFinder as implemented in IQ-TREE v. 1.6.7 (-MFP option) [52]. Following the selection of models of molecular evolution, we conducted 20 individual ML tree searches on the partitioned supermatrix (10 with a random starting tree and 10 with an IQtree-generated parsimony starting tree) (electronic supplementary material, §9).

To further evaluate relationships that were incongruent between the ML analysis on the concatenated supermatrix and the ASTRAL-III analysis, we performed four-cluster likelihood mapping (FcLM) on the concatenated supermatrix using IQ-TREE v. 1.6.7. In addition to the FcLM, we performed three series of permutation tests, a strategy previously used by Misof *et al*. [10]. In short, these tests were designed to evaluate potential confounding signal arising from amino acid composition bias, non-stationarity and/or the distribution of missing data through the creation and analysis of permuted datasets (full details in electronic supplementary material, §10).

### (e) Targeted enrichment analyses

Raw read pairs passing the CASAVA high-chastity filter were merged following Rokyta *et al*. [53] and library adapters were trimmed. We then followed the pipeline established by Breinholt *et al*. [54] for assembly and orthologue prediction. In short, we assembled each locus using an iterative-baited assembly process, where reads are mapped to reference loci and then assembled in local assemblies with Bridger [55]. We determined orthology by searching each locus against the reference genome for *Stenopsyche tienmushanensis* using BLAST [56]. If the locus generated hits to the reference genome on more than one contig with a bit score >90% of the primary hit bit score, or if the reciprocal BLAST search matched a different locus from the species sampled, the gene was removed from further analysis.

We then translated the alignments by predicting open reading frames in each locus using a custom script in Python 2.7 [57] and chose the reading frame that included no stop codons in any taxon. In each instance, this was unambiguous, with only a single reading frame without a stop codon across taxa.

### (f) Combined analyses

While the targeted enrichment probes were designed from the trichopteran transcriptomes, only portions of the original transcriptome assemblies were included in the final orthologue set. Thus, when combining datasets, we first identified loci that overlapped in both datasets. To do this, we used reciprocal blastp searches to identify putative overlapping orthologues. We aligned each orthologue with MAFFT v. 7.475 using the L-INS-i algorithm [44]. Following orthologue prediction, we generated trees for each gene. Each gene tree and corresponding alignments were inspected by hand. We identified two types of orthology mismatches (i) when taxa derived from transcriptome data and taxa derived from targeted enrichment data formed separate clades or (ii) when members of a family were polyphyletic and split among multiple clades. Such ortholog misassignments were removed from further analyses.

We concatenated the individual genes of the combined data into a supermatrix using FASconCAT v. 1.0 [47]. We then selected a partitioning scheme and models in IQ-TREE v. 1.6.7 using the -MERGE+MFP option [48,52]. Using this model, we conducted 30 individual tree searches in IQ-TREE (15 with a random starting tree and 15 with a parsimony starting tree) and selected the best tree for further analysis. We used the -bb option in IQ-TREE with 1000 replicates to estimate ultrafast bootstrap support and used the -bnni option to correct for potential overestimation of support in the presence of model violations. We also repeated the FcLM analyses on the combined dataset to evaluate the source of bias or signal regarding the relationships among the families of free-living (Rhyacophilidae and Hydrobiosidae) and tortoise-case-making (Glossosomatidae) caddisflies (electronic supplementary material, §10).

### (g) Molecular dating, ancestral state reconstructions and diversification analyses

We selected 33 fossils (32 Trichoptera and 1 Lepidoptera) to calibrate our tree (electronic supplementary material, table S6, §12). We then performed two dating analyses in MCMCtree [58,59], one with uniform priors and another with truncated Cauchy priors. The general methods follow Kawahara *et al*. [41]. In short, we estimated Hessian matrices on the aligned amino acids using the LG substitution model and five rate categories. We used CODEML to estimate empirical base frequencies. We then used the estimated Hessian matrix with a fixed topology from the best maximum likelihood tree (combined transcriptome and targeted enrichment) as input to MCMCtree. We ran four analyses for each set of node calibration priors (uniform and Cauchy). We additionally estimated the time priors for each analysis without the dataset to ascertain the informativeness of the priors (electronic supplementary material, figures S4 and S5). We removed the first 200 000 iterations as burnin and checked each run for convergence using Tracer v. 1.7 [60] and by plotting the posterior mean ages and the lower and upper bounds for the 95% credibility intervals for each of the runs against each other (electronic supplementary material, figures S6 and S7). Converged runs were then combined (full details in electronic supplementary material, §11).

ASRs for larval and pupal morphology were performed on the ML trees generated from the transcriptome-only and the combined datasets. Character matrices were generated for the following discrete characters: (i) larval construction behaviour, coded with five states (fixed retreat, purse case, tortoise case, no structure (i.e. free-living) and tube case); (ii) pupal case construction, coded with five states (dome-shaped enclosure, fixed silken larval tube case, modified purse case, newly constructed rigid tube case and modified larval tube case); (iii) cocoon construction, coded with three states (semi-permeable cocoon, permeable cocoon and silk-lined tube case); (iv) lentic versus lotic with two states (lentic and lotic). We used Phytools 2.1.1 [61] to conduct all ASRs, with ‘ancr’ and ‘simmap’. We first performed model selection to estimate the best transition models among both the built-in models in phytools and four custom unordered models (full details in electronic supplementary material, §13). We then estimated the ancestral states with ‘ancr’ using model averaging across all models. We additionally ran ‘make.simmap’ with the best-fit model and nsim set to 1000. For the combined dataset, we ran reconstructions on the 25 best ML trees to evaluate the sensitivity of the ancestral states to the topology.

We estimated diversification rates with both MiSSE [62] and BAMM [31]. In BAMM, we used two different strategies to estimate rates. For the first, we estimated diversification rates using the ultrametric tree derived from the MCMCtree dating analysis (figure 1) and accounted for unequal taxon sampling using lineage-specific sample proportions at the family level (electronic supplementary material, table S11). For the second, we used the stochastic polytomy resolution method implemented in TACT [32] to add unsampled taxa to the tree as this method has been shown to be less biased for inferring diversification rates than correction with lineage-specific sampling proportions [32]. We used the complete known taxonomy for Trichoptera as recorded in the Trichoptera World Checklist [5], resulting in a tree with 16 688 tips. For both datasets, we ran four separate diversification analyses in BAMM for 10 million generations. For each dataset, we used BAMMtools in R [63] to plot the log-likelihood against the generation number to assess burnin and subsequently removed the first 20% of each run where the log-likelihood reached an asymptote. We then estimated the effective sample size (ESS) using the R package coda. For each analysis, we then generated both the credible shift set and the maximum shift credibility in BAMMtools and compared the output from the four runs on each dataset to ensure convergence. We also used MiSSE as implemented in the hisse R package [64] to estimate diversification rates. We first used the generateMiSSEGreedyCombinations() function with max.param = 12 to generate a set of model combinations and then selected the best-fit model from those combinations using the MiSSEGreedy() function with the global sampling fraction (*f*) set to 0.013. We then performed 50 maximum likelihood optimizations for the best model from the previous step (i.e. the two-rate model) and plotted the most likely reconstruction with the MarginReconMiSSE() and plot.misse.states() functions. Lastly, we estimated the expected number of transitions by multiplying the transition rate (*q*) by the sum of the branch lengths in the phylogeny. To determine whether uncertainty in tree shape affected the results of the diversification analyses, we generated dated topologies for the 25 best maximum likelihood trees using treePL [65] and the same calibrations used in the MCMCtree analyses (full details in electronic supplementary material, §11). We then re-estimated diversification rates on each dated topology with MiSSE using the same steps described above.

## Data Availability

Sequence data are available on NCBI under the 1KITE umbrella BioProject PRJNA183205 (species-specific accessions are available in table S1). Supplementary tables and supplementary archives are available on Dryad [66]. Supplementary material is available online [67].
